# Thiazolidinediones enhance vascular endothelial growth factor expression and induce cell growth inhibition in non-small-cell lung cancer cells

**DOI:** 10.1186/1756-9966-29-22

**Published:** 2010-03-10

**Authors:** Takayuki Yoshizaki, Wataru Motomura, Sachie Tanno, Shima Kumei, Yumiko Yoshizaki, Satoshi Tanno, Toshikatsu Okumura

**Affiliations:** 1 Innovation Center, Kagoshima University, 1-21-40 Korimoto, Kagoshima 890-0065, Japan; 2 Department of General Medicine, Asahikawa Medical College, 2-1-1-1 Midorigaoka-Higashi, Asahikawa, Hokkaido 078-8510, Japan; 3 Department of Microbiology and Immunochemistry, Asahikawa Medical College, 2-1-1-1 Midorigaoka-Higashi, Asahikawa, Hokkaido 078-8510, Japan; 4 Department of Biochemical Science and Technology, Kagoshima University, 1-21-24 Korimoto, Kagoshima 890-0065, Japan

## Abstract

**Background:**

It is known that thiazolidinediones are involved in regulating the expression of various genes, including the vascular endothelial growth factor (VEGF) gene via peroxisome proliferator-activated receptor γ (PPARγ); VEGF is a prognostic biomarker for non-small-cell lung cancer (NSCLC).

**Methods:**

In this study, we investigated the effects of troglitazone and ciglitazone on the mRNA expression of VEGF and its receptors in human NSCLC cell lines, RERF-LC-AI, SK-MES-1, PC-14, and A549. These mRNA expressions were evaluated by quantitative real-time reverse transcription-polymerase chain reaction (RT-PCR) analysis. We also studied the effect of Je-11, a VEGF inhibitor, on the growth of these cells.

**Results:**

In NSCLC cells, thiazolidinediones increased the mRNA expression of VEGF and neuropilin-1, but not that of other receptors such as fms-like tyrosine kinase and kinase insert domain receptor-1. Furthermore, the PPARγ antagonist GW9662 completely reversed this thiazolidinedione-induced increase in VEGF expression. Furthermore, the addition of VEGF inhibitors into the culture medium resulted in the reversal of thiazolidinedione-induced growth inhibition.

**Conclusions:**

Our results indicated that thiazolidinediones enhance VEGF and neuropilin-1 expression and induce the inhibition of cell growth. We propose the existence of a pathway for arresting cell growth that involves the interaction of thiazolidinedione-induced VEGF and neuropilin-1 in NSCLC.

## Background

Peroxisome proliferator-activated receptor γ (PPARγ) belongs to a family of ligand-activated transcription factors. PPARγ is an intracellular sensor for fatty acids and fatty acid derivatives, which in turn act as endogenous ligands for PPARγ. PPARγ and its ligand activators regulate several lipid and glucose metabolism pathways [1].

In humans, PPARγ is expressed in multiple tissues, including the breast, colon, prostate, lung, placenta, and pituitary tissues [2-5]. PPARγ activation is antiproliferative by virtue of its differentiation-promoting effects. For example, ligands activating PPARγ were effective in arresting the growth of dedifferentiated tumor cells in multiple tumor types [2,4-9], and they promoted differentiation of tumor cells and inhibited spontaneous metastasis in a xenograft model [7]. However, the mechanism by which PPARγ arrests growth has not been completely clarified.

PPARγ is a molecular target for thiazolidinediones (TZDs), a class of insulin-sensitizing agents, such as troglitazone, ciglitazone, pioglitazone, and rosiglitazone. It is known that TZDs are involved in regulating the expression of various genes, including the genes encoding vascular endothelial growth factor (VEGF) and its receptors. VEGF (also called VEGF-A) is one of the most potent angiogenic factors, playing a key role in the physiological regulation of endothelial cell growth. It has been reported that rosiglitazone represses VEGF expression via a PPARγ-responsive element in the VEGF gene promoter [10] and that pioglitazone reduces VEGF expression [11]. On the other hand, there are several contradictory reports stating that thiazolidinediones increase VEGF expression [12-19]. This difference in results may be because of the different cell type used in the study. But it is unclear whether these conflicting results are because of any mechanism.

Currently, lung cancer is the most frequent cause of cancer-related deaths in the developed world, and the chief histological type (affecting about 80% of lung cancer patients) is non-small-cell lung cancer (NSCLC). With the advent of partially effective but potentially toxic adjuvant chemotherapy, it has become important to find biomarkers for identifying patients with the highest likelihood of recurrence, and who will benefit most from the adjuvant chemotherapy. In the past several decades, many papers have reported molecular markers or proteins that may have prognostic significance in NSCLC. One such study reported that increased VEGF expression has consistently been shown to affect NSCLC outcome [20]. Thus, VEGF is thought to be a molecular marker and therapeutic target in managing NSCLC.

Although TZDs arrest cell growth, including the growth of NSCLC cells, the relationship between its anti-tumor effect of and the regulation of VEGF expression is unknown. Therefore, the aim of this study was to investigate whether TZDs up- or down-regulate the expression of VEGF-A and its receptors in NSCLC and whether these VEGF-receptor interactions influence cell growth.

## Methods

### Human NSCLC cell lines

Lung squamous cell carcinoma line RERF-LC-AI, lung adenocarcinoma cell lines PC-14 and A549 were obtained from the RIKEN BioResource Center, Ibaraki, Japan. Lung squamous cell carcinoma line SK-MES-1 was purchased from DS Pharma Biomedical, Osaka, Japan. The RERF-LC-AI cells were cultured in a Minimal Essential Medium (MEM) (Sigma-Aldrich, St. Louis, MO, USA) supplemented with 10% fetal bovine serum (Invitrogen, Carlsbad, CA, USA). The SK-MES-1 cells were cultured in MEM containing 10% fetal bovine serum and 1% non-essential amino acids (Invitrogen, Carlsbad, CA, USA). The PC-14 cells were cultured in RPMI1640 medium (Invitrogen, Carlsbad, CA, USA) supplemented with 10% fetal bovine serum. The A549 cells were cultured in Dulbecco's Modified Eagle's Medium (DMEM) (Invitrogen, Carlsbad, CA, USA) supplemented with 10% fetal bovine serum. The cells were incubated at 37°C in a humidified atmosphere of 5% CO_2 _in air.

### Chemicals

Troglitazone was kindly provided by Daiichi Sankyo (Tokyo, Japan). Ciglitazone, GW9662, Je-11, and JNK Inhibitor II were purchased from Calbiochem (La Jolla, CA, USA); U0126 was purchased from Promega (Madison, WI, USA); and SB 202190 from Sigma-Aldrich (St. Louis, MO, USA). These chemicals were dissolved in dimethyl sulfoxide (DMSO) with a final concentration of 0.1% DMSO in the culture medium.

### Quantitative real-time RT-PCR analysis

Total RNA was extracted from the RERF-LC-AI, SK-MES-1, PC-14, or A549 cells by using TRIzol reagent (Invitrogen, Carlsbad, CA, USA). Complementary DNA was synthesized using 0.1 μg of total RNA and random primers, with the RETROscript kit (Ambion, Austin, TX, USA). Quantitative real-time RT-PCR analysis was performed using the Applied Biosystems 7300 Real-Time PCR System and the TaqMan Gene Expression Master Mix, according to the manufacturer's specifications (Applied Biosystems, Foster City, CA, USA). TaqMan probes for human VEGF-A (Hs00173626_m1), KDR (Hs00176676_m1), Flt-1 (Hs00176573_m1), NRP-1 (Hs00826129_m1), hypoxia-inducible factor 1α (HIF-1α) (Hs00153153_m1), and PPARγ coactivator-1α (PGC-1α) (Hs00173304_m1) were also purchased from Applied Biosystems. To normalize the relative expression of the genes of interest, eukaryotic 18S rRNA (Hs99999901_s1, X03205.1) was used as an endogenous control. All experiments were performed in triplicate.

### Western blot analysis

The protein extracts (5 μg) obtained from the PC-14 cells were separated using 5-20% sodium dodecyl sulfate polyacrylamide gel electrophoresis (SDS-PAGE). After electrophoresis, the proteins were transferred to a polyvinylidene difluoride (PVDF) membrane (Millipore, Bedford, MA, USA) and blocked overnight in BlockAce (Dainippon Sumitomo Pharma, Osaka, Japan) at 4°C. The proteins were then reacted with primary polyclonal antibodies against human β-actin (#4967; Cell Signaling Technology, Beverly, MA, USA), VEGF (ab46154; Abcam, Cambridge, UK), Phospho-MAPK Family (#9910; Cell Signaling Technology), or MAPK Family (#9926; Cell Signaling Technology) at 4°C overnight, washed with Tris Buffered Saline Tween (TBST), reacted with secondary polyclonal antibodies against rabbit IgG (Chemicon International, Temecula, CA, USA) for 1 h, and washed again with TBST. After being reacted with horseradish peroxidase-conjugated anti-rabbit IgG, the immune complexes were visualized using ECL Plus detection reagents (GE Healthcare, Waukesha, WI, USA) and the Luminescent Image Analyzer LAS-3000 (Fujifilm, Tokyo, Japan).

### Cell growth assay

The cell number was determined by performing the WST-1 assay using the Cell Counting Kit (Dojindo, Kumamoto, Japan), as we have reported previously [9]. Briefly, 100 μl of the PC-14 cells, at a concentration of 8 × 10^4 ^cells/ml were seeded on a 96-well cell culture plate (Corning, Corning, NY, USA). After 24 h, each well was incubated with various concentrations of troglitazone and Je-11 for 0, 24, or 48 h. After each incubation period, cell growth was determined using the Cell Counting Kit and a model 680 microplate reader (Bio-Rad Laboratories, Richmond, CA, USA) at a wavelength of 405 nm.

### Statistical analysis

Data are expressed as mean (SD). Statistical analysis was performed either by one-way analysis of variance and subsequent Tukey multiple comparison procedure, or by two-way analysis of variance with subsequent Bonferroni post-test; all of these were performed using the GraphPad Prism Software (version 4). *P *< 0.05 was considered statistically significant.

## Results

**Figure 1:**
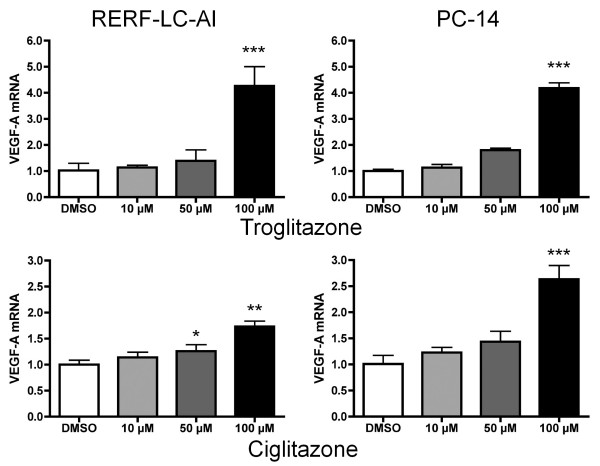
**Effect of TZDs on VEGF-A mRNA expression in lung cancer cell lines**. RERF-LC-AI (left panel) and PC-14 (right panel) cells were treated with 0, 10, 50, or 100 μM of troglitazone (upper panel) or ciglitazone (lower panel). The culture medium contained 0.1% DMSO to maintain the same conditions throughout the experiments. After 24 h of treatment, specific mRNA was quantified using real-time PCR. Data were normalized relative to the level of 18S rRNA, and expressed as mean (SD) (n = 3). *P < 0.05, **P < 0.01, ***P < 0.001 vs. vehicle control.

First, we determined whether troglitazone affects the expression of VEGF-A and its receptors, fms-like tyrosine kinase (FLT-1/VEGFR1), kinase insert domain receptor 1 (KDR/VEGFR2), and neuropilin-1 (NRP-1) in the human lung cancer cell lines, RERF-LC-AI, SK-MES-1, PC-14, and A549 (Table 1). In these cell lines, we found that troglitazone had a dose-dependent effect on the expression of VEGF-A mRNA. To further prove that troglitazone enhances VEGF-A expression in lung cancer cells, we studied the effects of ciglitazone on the expression of VEGF-A mRNA in the RERF-LC-AI and PC-14 cells. Ciglitazone enhanced the expression of VEGF-A mRNA in both cell lines; however, it was less effective than troglitazone (Figure 1). The mRNA expression of its receptors, KDR and FLT-1, was hardly affected; however, mRNA expression of NRP-1, which is thought to be a receptor of the VEGF-A splicing variant VEGF165 [21], was affected in a dose-dependent manner. In addition, the level of FLT-1 and KDR mRNA expression in the all cell lines were extremely low (threshold cycle values of these mRNAs were around 34-37 cycles; data not shown), or not detected (N.D.). We also investigated the mRNA expression of transcription factor HIF-1α, a known regulating factor of VEGF-A [22,23], and transcriptional coactivator PGC-1α (Table 1). Our results indicate that troglitazone significantly enhanced HIF-1α expression in the RERF-LC-AI, SK-MES-1, and PC-14 cells (Table 1). On the other hand, the expressions of PGC-1α mRNA in the RERF-LC-AI and SK-MES-1 cells were not affected by troglitazone, and PGC-1α mRNA in the PC-14 cells was not detected. These results indicate that, in NSCLC, troglitazone enhances VEGF-A mRNA expression by increasing HIF-1α expression, and that the VEGF-A receptor is mainly NRP-1. We hypothesize that the interactions of VEGF-A and NRP-1 directly affect cell growth, because the arrest of cell growth by TZDs has been widely reported.

**Table 1:** Relative mRNA expression levels of VEGF-A, its receptors, transcription factor HIF-1α, and transcriptional coactivator PGC-1α.

Troglitazone (μM)		VEGF-A	FLT-1	KDR	NRP-1	HIF-1α	PGC-1α
RERF-LC-AI (Squamous cell carcinoma)	DMSO	1.00 ± 0.28	1.00 ± 0.13	N.D.	1.00 ± 0.03	1.00 ± 0.16	1.00 ± 0.20
	10	1.14 ± 0.08	1.08 ± 0.43		1.00 ± 0.18	1.24 ± 0.31	0.95 ± 0.20
	50	1.39 ± 0.42	0.97 ± 0.48		1.03 ± 0.45	1.27 ± 0.23	0.82 ± 0.05
	100	4.26 ± 0.74 **	1.23 ± 0.18		5.79 ± 0.48***	1.35 ± 0.26	0.92 ± 0.10
							
SK-MES-1 (Squamous cell carcinoma)	DMSO	1.00 ± 0.06	N.D.	1.00 ± 0.24	1.00 ± 0.04	1.00 ± 0.23	1.00 ± 0.41
	10	1.21 ± 0.17		1.29 ± 0.26	1.09 ± 0.11	1.40 ± 0.66	1.00 ± 0.26
	50	1.81 ± 0.18**		0.60 ± 0.05	1.07 ± 0.04	3.07 ± 0.32***	1.09 ± 0.22
	100	3.34 ± 0.16***		0.49 ± 0.15*	1.42 ± 0.06***	3.13 ± 0.11***	0.85 ± 0.06
							
PC-14 (Adenocarcinoma)	DMSO	1.00 ± 0.07	N.D.	N.D.	1.00 ± 0.05	1.00 ± 0.05	N.D.
	10	1.13 ± 0.12			0.98 ± 0.11	1.29 ± 0.09**	
	50	1.80 ± 0.08			1.29 ± 0.47	1.39 ± 0.08**	
	100	4.18 ± 0.21***			1.68 ± 0.24*	1.35 ± 0.09**	
							
A549 (Adenocarcinoma)	DMSO	1.00 ± 0.05	N.D.	N.D.	1.00 ± 0.12	1.00 ± 0.23	1.00 ± 0.10
	10	1.06 ± 0.11			0.89 ± 0.05	1.40 ± 0.66	1.16 ± 0.28
	50	1.90 ± 0.32***			1.35 ± 0.42	3.07 ± 0.32***	1.95 ± 0.44**
	100	2.10 ± 0.16***			1.04 ± 0.12	3.13 ± 0.11***	1.36 ± 0.06

Data were normalized relative to the level of 18S rRNA, and expressed as mean (SD) of 3 experiments. *P < 0.05, **P < 0.01, ***P < 0.001 vs. vehicle control.

**Figure 2:**
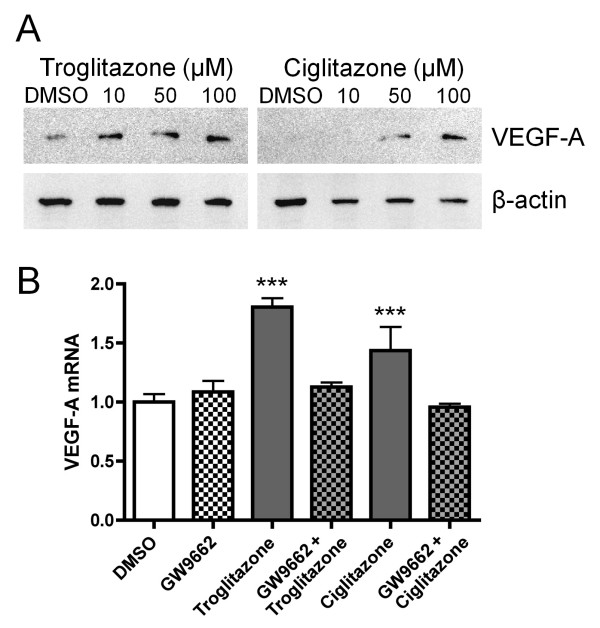
**The expression of VEGF-A protein and PPARγ dependent pathway**. A. PC-14 cells were treated with 0, 10, 50, or 100 μM troglitazone or ciglitazone and 48 h after treatment the expression of VEGF-A protein was measured by western blot analysis. B. PC-14 cells were treated with or without GW9662 (20 μM), a PPARγ inhibitor, for 1 h before they were exposed to troglitazone or ciglitazone (50 μM each). After 24 h of thiazolidinedione treatment, the relative expression of VEGF-A mRNA was evaluated using real-time PCR. Data are expressed as mean (SD) (n = 3). ***P < 0.001 vs. vehicle control.

To clarify the correlation between the interaction of VEGF-A and its receptor NRP-1, and cell growth inhibition by troglitazone, PC-14 cells were used for the following experiment. Because the expressions of FLT-1 and KDR mRNA were not detected in the PC-14 cells. Western blot analysis showed that VEGF-A protein levels varied with TZD levels in a dose-dependent manner (Figure 2A). The results were consistent with those obtained by RT-PCR analysis. GW9662, a PPARγ antagonist, completely blocked the TZD-induced expression of VEGF-A mRNA through a PPARγ-dependent pathway in the PC-14 cells (Figure 2B). These results indicate that the TZDs--troglitazone and ciglitazone--induce the expression of VEGF-A mRNA and protein and that this induction depends on PPARγ activation.

**Figure 3:**
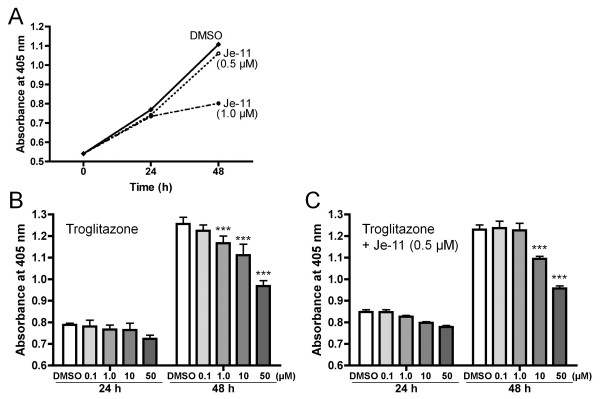
**Effect of a VEGF inhibitor with several concentrations of troglitazone on cell proliferation**. A. PC-14 cells were treated with either 0, 0.5, or 1.0 μM Je-11 and cell numbers were determined after 0, 24, and 48 h. PC-14 cells were treated with either 0, 0.1, 1.0, 10, or 50 μM troglitazone containing either 0 μM Je-11 (B) or 0.5 μM Je-11 (C) and cell numbers were determined 24 h and 48 h after treatment. Data are expressed as mean (SD) (n = 6). ***P < 0.001 vs. vehicle control.

We investigated the effects of VEGF-A on cell growth by using the VEGF inhibitor Je-11. Je-11 directly binds to VEGF and acts as an inhibitor of VEGF-stimulated autophosphorylation [24]. It was found that 0.5 μM of Je-11 had a marginal effect, whereas 1.0 μM had serious effects on cell growth (Figure 3A). Thus, we investigated whether Je-11 affects troglitazone-induced VEGF-A-mediated cell growth arrest (Figure 3B, C). Interestingly, we found that 1.0 μM of troglitazone could not arrest cell growth in the presence of 0.5 μM Je-11. Although there have been no reports suggesting that the binding of VEGF-A and Je-11 causes inhibition of VEGF-A (VEGF165) and NRP-1, our result suggests that the growth inhibition of the PC-14 cells by troglitazone depends on VEGF-A and its receptors in these cells.

**Figure 4:**
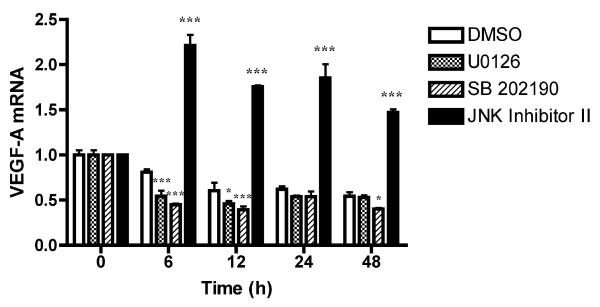
**Effect of the MAPK inhibitors on the expression of VEGF-A mRNA**. PC-14 cells were treated with 10 μM of each inhibitor for MEK (U0126), p38 (SB 202190), and JNK (JNK Inhibitor II), and specific mRNA was quantified 0, 6, 12, 24, and 48 h after treatment by using real-time PCR. Data were normalized relative to the level of 18S rRNA and expressed as mean (SD) (n = 3). *P < 0.05, ***P < 0.001 vs. vehicle control.

**Figure 5:**
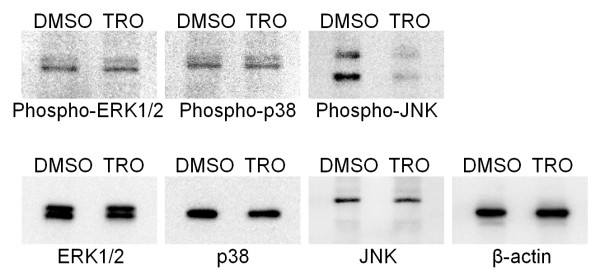
**Effect of troglitazone treatment on levels of phosphorylated MAPKs**. PC-14 cells were treated with 100 μM troglitazone (TRO) or vehicle and 24 h after treatment, phosphorylated-MAPK protein levels of ERK1/2, p38, and JNK were measured by western blot analysis.

Mitogen-activated protein kinases (MAPKs) are key participants in cell proliferation, survival, and differentiation. Hence, we investigated the role of MAPKs in the mechanism by which troglitazone induces the expression of VEGF-A mRNA. The MAPK family is composed of 3 distinct protein kinases MEK-ERK1/2, p38, and c-Jun N-terminal kinase (JNK). To clarify whether the signaling of each MAPK is involved in the enhancement of VEGF-A expression by troglitazone, we examined the effects of the inhibitors of MEK (U0126), p38 (SB 202190), and JNK (JNK Inhibitor II). We found that enhanced VEGF-A expression was required for the inhibition of JNK phosphorylation and that VEGF-A enhancement was slightly arrested when using the MEK inhibitor U0126 and the p38 inhibitor SB 202190 compared to vehicle control (Figure 4). Additionally, Figure 5 indicates that phosphorylated-JNK levels were clearly reduced in PC-14 cells treated with troglitazone, whereas other phosphorylated- and non-phosphorylated MAPKs remained at the same level. These results indicate that troglitazone-induced VEGF-A expression is negatively regulated by the JNK signaling pathway.

## Discussion

In this study, we showed that TZDs increase the mRNA expression of VEGF-A and NRP-1 but not that of FLT-1 and KDR in NSCLC cells. We also showed that GW9662, a PPARγ antagonist, completely reverted the TZD-induced expression of VEGF-A mRNA to the original level and that this was accompanied by the expression of transcriptional factor HIF-1α. VEGF-A expression has been reported to be regulated by transcription factor HIF-1α [22,23]. Recently, it has been reported that the transcriptional coactivator PGC-1α regulates VEGF expression by an HIF-1α independent pathway [25]. Our results indicate that troglitazone significantly enhances VEGF-A expression in a HIF-1α-dependent manner.

Western blot analysis showed that the level of VEGF-A proteins also increased in the presence of TZDs. Therefore, we also studied the effect of the VEGF inhibitor Je-11. Recently, it has been reported that anti-VEGF monoclonal antibodies significantly arrest cell growth in SK-MES-1, a squamous cell carcinoma cell line from the lung [26]. However, an interesting finding of our study was that the inhibition of VEGF by Je-11 partially blocked the troglitazone-induced growth inhibition in NSCLC cells, whereas FLT-1 and KDR are still present albeit in very small amounts. Because NRP-1 binds only to the VEGF-A isoform VEGF165 [22], these results suggest that growth is arrested by the interaction of VEGF165 and NRP-1. In addition, our results showed that troglitazone reduces phosphorylated-JNK levels and inhibiting the phosphorylation of JNK is necessary for inducing the expression of VEGF-A mRNA. Similarly, it has been reported that TZD inhibits the proliferation of human NSCLC NCI-H23 cells and that these effects are associated with ERK1/2 activation and SAPK/JNK deactivation [27]. Although we did not detect ERK1/2 activation, JNK deactivation was observed at 24 h after TZD treatment (Figure 5). These differences might be attributed to the concentration of TZD and the type of cell line. Further, JNK inhibitors upregulated the expression of VEGF mRNA at all time points after treatment, but MEK inhibitor and p38 inhibitor did not affect the expression of VEGF-A mRNA at 24 h after treatment, as compared to the expression in the vehicle control. Taken together, these results indicate that TZD-induced VEGF-A expression is negatively regulated mainly by the JNK pathway.

VEGF is a major angiogenic factor that stimulates the proliferation and migration of endothelial cells. Four VEGF isoforms composed of 121, 165, 189, and 206 amino acids can be synthesized by alternative splicing of VEGF mRNA. The larger isoforms (VEGF189 and VEGF206) are cell-associated and bind to glycosaminoglycans, whereas the smaller isoforms (VEGF121 and VEGF165) are secreted into the extracellular matrix [23]. Recently, it was reported that VEGF189 is the major VEGF-A isoform present in NSCLC cells, and the expression of VEGF189 mRNA, in NSCLC cells, was 5 to 10 times higher than that of VEGF165 mRNA [28]. VEGF165 is mainly secreted, whereas VEGF189 is cell-associated and is almost completely sequestered in the extracellular matrix [23]. These VEGF isoforms probably have different functions in cancer tissues. Although several types of tumor cells express VEGF-A and its receptors, the VEGF-A receptor neuropilin-1 (NRP-1) is only expressed in the pancreatic carcinoma cell lines Panc-1 and MIA PaCa-2 [29]. Because NRP-1 only binds to VEGF165, one of the several isoforms of VEGF-A [21], it is possible that the binding of VEGF165 to NRP-1 causes cell progression in these pancreatic carcinoma cells. Furthermore, the results of studies on VEGF inhibition using Je-11 suggested that VEGF enhances cell proliferation (Figure 3A). However, the inhibition of VEGF by Je-11 partially relieved the TZD-induced cells from growth arrest. Therefore, we believe that TZD treatment cause the growth arrest of NSCLC cells by the mechanism containing VEGF-A (VEGF165) and NRP-1 interaction.

High VEGF expression has been reported to be associated with poor prognosis in patients with breast carcinoma [30], prostate carcinoma [31], melanoma [32,33], and lung carcinoma [20]. Thus, VEGF is a prognostic biomarker for NSCLC. On the other hand, lung cancer risk among subjects administered with TZDs is reduced by 33% [34] and in vitro studies indicate that TZDs inhibit the growth of NSCLC cells [27,35]. Purified VEGF189 and VEGF165 induced cell progression in human umbilical vascular endothelial cells (HUVEC), the human metastatic breast cancer cell line MDA-MB-231, and the human pancreatic carcinoma cell line Panc-1 [36]. These reports indicated that one of the mechanisms as an anti-cancer effect of TZDs was depressing the VEGF expression.

However, some reports contradict the inductive effect of TZDs on VEGF [12-19], and this was also observed in the present study. Our results indicate that the interaction of the induced VEGF and NRP-1 may inhibit the growth of NSCLC cells. Taken together, these results suggest that rather than being a growth factor for NSCLC cells, troglitazone-induced VEGF may mediate cell growth arrest.

It has been recently reported that the mechanism of VEGF action is complicated [37]. Deletion of myeloid-cell VEGF-A in multiple subcutaneous isograft models and in an autochthonous transgenic model of mammary tumorigenesis resulted in accelerated tumor progression; this process was accompanied by less overall tumor cell death and decreased tumor hypoxia. Administration of TZD to a lung cancer patient induces VEGF expression and prevents the maturation of the surrounding blood vessels, thereby leading to tumor suppression by hypoxia and lack of nutrition. Further, in this study, we showed that TZD-induced VEGF expression inhibited the growth of tumor cells. We think that both these effects prolong the survival of the lung cancer patients. On the basis of these results, TZD can be used as an effective anti-cancer agent for the treatment of lung or other cancer patients with high VEGF expression receptivity toward TZD.

## Conclusions

In the present study, we report the existence of a new pathway for arresting cell growth that involves the interaction of troglitazone-induced VEGF and NRP-1 in NSCLC cells. This suggests that TZDs may be effective anti-cancer agents, and it may be possible to develop a new anti-cancer therapy if the mechanisms underlying these anti-cancer effects are better understood.

## Competing interests

The authors declare that they have no competing interests.

## Authors' contributions

TY carried out the molecular genetic studies and wrote the manuscript; WM, SK, and YY carried out the immunoassays and statistical analysis; ST and TO participated in the design of the study. All authors read and approved the final manuscript.
